# Contribution of major economies to global GDP and product reallocation around the world

**DOI:** 10.1016/j.heliyon.2023.e23909

**Published:** 2023-12-19

**Authors:** Maria Llop

**Affiliations:** Universitat Rovira i Virgili and ECO-SOS, Department of Economics, Avinguda Universitat 1, 43204 Reus, Catalonia Spain

**Keywords:** Global GDP, Contribution to GDP, Product reallocation, Multi-country input-output model

## Abstract

Economic globalization has led to production increasingly becoming concentrated in certain regions and countries of the world. This article develops an accounting framework to provide the trends for the contribution of countries to global gross domestic production (GDP). In particular, the method transforms the multiregional input-output model to quantify the relative importance of individual economies to world GDP. The proposal uses a world input-output database that distinguishes between three main economic areas: China, the United States of America and the European Union. The results suggest that identical changes in sectoral demand asymmetrically modify the relative contributions from individual economies, with China showing the highest positive (negative) impact after an increase (decrease) in world demand. These findings suggest that a generalized economic expansion (contraction) is linked to a boost (decline) in the asymmetries of production across the world.

## 1 Introduction

Over the last hundred years, globalization has created a geographically concentrated production system in which certain areas of the planet now operate as engines of the entire world's gross domestic production (GDP). This agglomeration of production activity has led to new trends in economic geography in which global GDP is obtained by a relatively small number of regions that hold the world's economic performance in their hands. According to OECD statistics, in 1996 China's contribution to global GDP (adjusted by purchasing power parity) was 6.8 %, while that of the United States of America was 26.9 % and that of the OECD countries of the European Union (EU) was 28.5 %.^1^ By 2022, China's contribution was 22.3 %, while that of the United States was 21.4 % and that of the OECD countries of the EU was 18.9 % [1]. This means that these three regions jointly contributed 62.2 % to global GPD in 1996 and 62.6 % in 2022. Although the contribution from this economic area changed little between 1996 and 2022, there was a modification within the group as China's relative importance increased with respect to that of both the United States and the European Union.

From a global perspective, the distribution of world production is a central issue in the debate on the gap between rich and poor countries.^2^ Broadly speaking, there are two perspectives for understanding this gap, namely the classical dichotomy between economic convergence (i.e., a decreasing gap over time) and economic divergence (i.e., an increasing gap over time).^3^ Moreover, the policy relevance of product allocation worldwide is explained by its connection to global distributive aspects such as the distribution of income and welfare between countries.^4^

From a national perspective, a country's large contribution to global GDP is linked to a large contribution by that country to world trade, a certain degree of market power for its national products in the global markets, and the possibility of exerting influence on world prices.^5^ Moreover, a country's role in international forums and multinational organizations is largely determined by its economic significance.^6^ An easy way to identify that significance is to analyze the country's contribution to global production. Changes in the (individual) relative contribution to global GDP also enable local policymakers to judge whether the external context is providing economic advantages domestically. In other words, since the distribution of global production provides an overall picture of the wealth of individual economies, it also provides the option to judge each country's degree of success on the global scene.

The literature has examined the distance between income in rich and poor countries both theoretically and empirically. Within this line of research, one issue focuses on trends in global GDP, which are evaluated using various modeling techniques such as statistical analyses of the economic organization worldwide and projections for the future directions in world production.^7^ Another issue focuses on the consequences of the new production geography on international income distribution, with special emphasis on temporal variations in inequality between countries.^8^ Another line of research makes dynamic projections to determine the relative contributions of different countries and regions to future global production.^9^

In the last few decades, the development of world production statistics, and especially those related to input-output intersectoral and intercountry transactions, has enabled the construction of multiregional (multi-country) models to explain the determining factors of world production. By transforming this model, we can also determine the relative contribution of countries to global production. This transformation enables us to identify the mechanisms underlying the individual role (i.e., the individual position of countries) within the global production mosaic. This paper discusses these issues and adapts a worldwide version of a multi-country input-output model to define the production of individual economies in relative terms. By assuming changes in the model's exogenous components, we can quantify how far each country's relative GDP is modified by changes in economic scenarios.

Interregional input-output literature contains a huge number of contributions focused on the interconnections between regions. In particular, the interregional model has the option of quantifying production impacts emerging in one part (country/region) of the model but originating in another (country/region).^10^ Like many other authors [[23], [24], [25]], proposed the pioneering input-output interregional contributions. Since these initial models, numerous studies have been published, thereby demonstrating the great explanatory potential of this research field.^11^

The aim of this paper is to examine the underlying mechanisms that determine the role played by countries within the global production system. This will help to further our knowledge of the factors behind the economic importance of countries and their contribution to world GDP. Since the geographical configuration of economic activity and income distribution worldwide helps to shed light on the rich-country/poor-country debate and to better understand the gap between rich and poor countries and regions, the underlying factors affecting the position of individual economies are of crucial interest. This paper develops these aspects by using a (relatively simple) input-output approach which has an undeniable explanatory potential and clarifies some of the complex and intricate determinants of world production.

Although the multiregional input-output model has been used largely to describe production channels across regions and countries, to the best of my knowledge no paper has studied production issues in relative terms, while the relative income modelizations developed so far are circumscribed in the context of the social accounting matrix (SAM) models, which are an extension of the input-output (limited-to-production) model. Specifically, the SAM framework contains larger income channels since it includes institutional sectors (i.e., consumers and value added) in the determination of endogenous income. Among SAM analyses for studying relative incomes [30], presented a relative measure to investigate the distribution of the multiplier effects among the components of a model constructed for the Netherlands [31]; proposed a general and systematic analytical framework to define the SAM income distribution process and provided an application for the United States [32]; used the context of [31] to analyze redistributional impacts among various types of UK households and emphasized the role of the agricultural sector through the impact of agricultural policies [33]; identified several components of SAM income redistribution channels and presented an empirical application to the region of Catalonia (Spain) [34]; analyzed the distributive impacts of alternative agricultural policies using a SAM for Italy [35]; applied the relative income model to a SAM for the region of Extremadura (Spain); and for the Chinese province of Jiangsu [36], studied the distribution mechanism between tourism and non-tourism industries.

In this paper, the input-output model is transformed to provide a relative measurement of the contributions from individual countries or regions to global GDP. The (world-focused) method also evaluates how far the countries’ contributions to world production (or relative GDP) are affected by changes in the (exogenous) final demand of sectors. Since this approach includes a rich set of information, the outcomes rely not only on a worldwide perspective of production processes but also on specific implications for: i) each single country or region, and ii) each sector of production reflected in the model. The empirical application considers the three major contributors to world production (China, the United States, and the European Union) as well as the Rest of the World (ROW). By looking at the positions of the main economic blocks at the world level and their changes after global economic expansions, we are able to put forward a superficial analysis of world production statistics. Moreover, the novel input-output model used enables us to further examine the intersectoral and intercountry linkages behind the disparity in the economic importance of countries. All these issues enable us to go beyond a mere quantitative interpretation of statistics, by showing several determining factors of the economic importance of countries and the transmission channels of this influence across countries.

The rest of this paper is organized as follows. Section 2 describes the multiregional setting, derives measurements of the contributions of countries to production, and evaluates how changes in final demand affect the relative importance of countries. Section 3 describes the database used, while Section 4 shows the empirical results and discusses the main findings. Finally, Section 5 draws conclusions.

## 2 Modeling framework

### 2.1 Global GDP in the two-country case

The starting point for analyzing the contribution of countries to world production is the multiregional input-output framework, which explicitly captures the economic connections between countries throughout the sectoral inputs and outputs circulating worldwide. Compared with other partial-equilibrium methods, the (deterministic) input-output framework allows the production system to be comprehensively described by using a general-equilibrium view of the interregional and intersectoral transactions. Moreover, the approach used enables the production value chains to be completely covered by the analysis, resulting in a genuinely global model.

For the sake of simplicity, let us assume a world with two countries or regions (s and r). Goods produced there can be used either as intermediate inputs or final products. In both areas, the intermediate and final goods are either consumed within the area's borders or exported to the other. The input-output model characterizing this two-country system can be represented as follows:

(1) $$\left[\begin{array}{c} x_{s}\\ x_{r} \end{array}\right]=\left[\begin{array}{cc} A_{ss} & A_{sr}\\ A_{rs} & A_{rr} \end{array}\right]\left[\begin{array}{c} x_{s}\\ x_{r} \end{array}\right]+\left[\begin{array}{cc} y_{ss} & y_{sr}\\ y_{rs} & y_{rr} \end{array}\right]\left[\begin{array}{c} 1\\ 1 \end{array}\right]\text{,}$$

where $A=\left[\begin{array}{cc} A_{ss} & A_{sr}\\ A_{rs} & A_{rr} \end{array}\right]$ is a block matrix of the input-output structural coefficients, which are calculated by dividing the intermediate goods from sector i by the gross output of sector j. Note that this matrix includes all possible intercountry transactions: $A_{ss}$ and $A_{rr}$ include the sectoral transactions within each region or country, while $A_{sr}$ and $A_{rs}$ represent the sectoral transactions between regions or countries (from s to r and from r to s, respectively). In Equation (1), $X=\left[\begin{array}{c} x_{s}\\ x_{r} \end{array}\right]$ is the vector of gross output, $Y=\left[\begin{array}{cc} y_{ss} & y_{sr}\\ y_{rs} & y_{rr} \end{array}\right]$ is the matrix of intercountry final demand transactions, and $e=\left[\begin{array}{c} 1\\ 1 \end{array}\right]$ is a unitary column vector that adds up the elements in the rows of matrix Y.

Equation (1) can alternatively be written as:

(2) $$\left[\begin{array}{c} x_{s}\\ x_{r} \end{array}\right]={\left[\begin{array}{cc} {I-A}_{ss} & -A_{sr}\\ {-A}_{rs} & {I-A}_{rr} \end{array}\right]}^{-1}\left[\begin{array}{cc} y_{ss} & y_{sr}\\ y_{rs} & y_{rr} \end{array}\right]\left[\begin{array}{c} 1\\ 1 \end{array}\right]\text{.}$$

From this general production setting, gross domestic product is obtained by transforming expression (2) as follows:

(3) $$\left[\begin{array}{c} g_{s}\\ g_{r} \end{array}\right]={\left[\begin{array}{cc} v_{s} & 0\\ 0 & v_{r} \end{array}\right]\left[\begin{array}{cc} {I-A}_{ss} & -A_{sr}\\ {-A}_{rs} & {I-A}_{rr} \end{array}\right]}^{-1}\left[\begin{array}{cc} y_{ss} & y_{sr}\\ y_{rs} & y_{rr} \end{array}\right]\left[\begin{array}{c} 1\\ 1 \end{array}\right]\text{,}$$

where $G=\left[\begin{array}{c} g_{s}\\ g_{r} \end{array}\right]$ is the column vector of the sectoral value added in s ($g_{s}$) and r ($g_{r}$), respectively, and $V=\left[\begin{array}{cc} v_{s} & 0\\ 0 & v_{r} \end{array}\right]$ is the diagonal matrix of the sectoral value added ratios in relation to gross output in s and r, respectively. Compactly, Equation (3) can be written as:

$$G=V{\left(I-A\right)}^{-1}Ye\text{,}$$

where ${\left(I-A\right)}^{-1}$ is the matrix of input-output multipliers or Leontief inverse matrix in the multiregional approach.

The global framework described above directly determines the individual (national or regional) output (Equation (2)) and individual value added (Equation (3)). Total (global) values can easily be calculated by simply adding the individual values. For the two-country case, global output would be $x=x_{s}+x_{r}$ or, alternatively, x=e’X with e’ being a unitary row vector. Similarly, global GDP would be $g=g_{s}+g_{r}$ or, alternatively, g=e’G. Another advantage of using the multi-country approach is that both the origin and destination of economic impacts can be identified and numerically quantified because all the interregional flows of goods are explicit in the determination of the economic relationships.

### 2.2 Relative GDP in the two-country case

The multiregional framework can be adapted to define each country's contribution to global GDP.^12^ By transforming expression (4) to define relative values, the contribution to GDP is equal to:

(4) $$W=\frac{G}{e^{\prime }G}=\frac{V{\left(I-A\right)}^{-1}Ye}{e^{\prime }V{\left(I-A\right)}^{-1}Ye}\text{,}$$

where e’ adds up the elements in vector G so that $g=e^{\prime }G$ is the value of total GDP.^13^ In Equation (4), W is the *product contribution vector*, which contains two blocks, i.e., $w_{s}$ ($0<w_{s}<1$) and $w_{r}$ ($0<w_{r}<1$), which show sectoral contribution to global production in s and r, respectively. From this vector, the addition of sectors in each region (i and j, respectively) provides a total measurement of the region's contribution to global production: $W_{s}=\sum_{i=1}^{n}w_{s}$ and $W_{r}=\sum_{j=1}^{m}w_{r}$. This leads to the following structure for W:

$$W=\left[\begin{array}{c} W_{s}\\ W_{r} \end{array}\right]\text{,}$$

where the sum of the elements is necessarily equal to 1 (or, alternatively, 100 % of world GDP): i.e., $W_{s}+W_{s}=1$.

Equation (4) determines the relative importance of sectors and countries in function of the parameters of the input-output model, namely the elements in matrices A, V, and Y. Partial derivative of Equation (4) with respect to final demand matrix Y provides a quantification of how much the relative contribution to GDP changes when final demand is modified. Let us assume a change in Y, which modifies W as follows:

(5) $$dW=\frac{1}{e^{\prime }V{\left(I-A\right)}^{-1}Ye}\left[V-\frac{V{\left(I-A\right)}^{-1}Yee^{\prime }V}{e^{\prime }V{\left(I-A\right)}^{-1}Ye}\right]{\left(I-A\right)}^{-1}dY,=\frac{1}{e^{\prime }G}\left[V-\frac{Ge^{\prime }V}{e^{\prime }G}\right]{\left(I-A\right)}^{-1}dY=ZdY\text{.}$$

Equation (5) evaluates the changes in the contribution to GDP (dW) due to changes in the final demand matrix (dY). In this expression, Z is the *demand-to-product reallocation matrix*, which contains the changes in the relative positions (in global GDP) of the sectors of each country. This reallocation matrix provides insights into the underlying mechanisms operating in global production, since its elements ($z_{k,l}$) quantify by how much the relative position of sector k in country s or r is modified when there is a unitary inflow in the final demand of sector l in country s or r. For the two-region system described earlier, the structure of matrix Z is:

$$Z=\left[\begin{array}{cc} z_{ss} & z_{sr}\\ z_{rs} & z_{rr} \end{array}\right]\text{,}$$

where each element is a block containing the changes in the relative production of sectors due to exogenous inflows in the final demand of all other sectors. This is a complete portrait of all the possible effects, since matrix Z is made up of all the possible *vis-à-vis* connections reflected in the model. Note also that the individual elements in this matrix can either be positive, which shows an increase in a particular sector's contribution to total GDP, or negative, which shows a decrease in its contribution to total GDP.

At the aggregate level, it can be checked that the sum of the Z columns is null:

$$e^{\prime }Z=e^{\prime }\frac{1}{e^{\prime }G}\left[V-\frac{Ge^{\prime }V}{e^{\prime }G}\right]{\left(I-A\right)}^{-1}$$

$$=\frac{1}{e^{\prime }G}\left[e^{\prime }V-\frac{e'Ge'V}{e^{\prime }G}\right]{\left(I-A\right)}^{-1}=0\text{.}$$

This shows that the positive and negative values of the demand-to-product reallocation matrix balance out, so the changes in relative GDP, or the product reallocation process, can be understood as a zero-sum game.

## 3 Database

The empirical application is based on the latest version of the World Input-Output Database (WIOD), which contains data for the year 2014.^14^ The WIOD is a multiregional input-output table that originally comprised 56 sectors of production for 43 countries plus a residual Rest of the World (ROW). This database has been aggregated to individually show the three most important producers globally, i.e., China, the United States, and the European Union (EU),^15^ plus a residual Rest of the World to complete the world system. The aggregation of countries was done by including (i.e., adding) the intersectoral elements (k, l) of a country s to the corresponding Rest of the World account or to the European Union region.^16^ The 56 original sectors were divided into 25 activities: one agricultural sector, four energy activities, eight industries and twelve services. This aggregation was done by completely allocating all the original sectors of the WIOD to any of the new aggregated sectors so that no partial allocations to the compacted sectors were possible. The resulting database provided all the data (i.e., intercountry input-output transactions, sectoral value added and final demand) needed for the empirical analysis.

Applying this database to the model described in Section 2 involves 4 regional blocks, each of which has a number of sectors equal to i=j=25. This leads to an interregional structure made up of 4×25=100 accounts, so that matrix A has a dimension of 100×100, vector X of sectoral output has a dimension of 100×1, and matrix Y of intercountry final demand has a dimension of 100×12. The number of columns in the final demand matrix is equal to 4×3=12 since each of the 4 regions contains 3 demand elements (private consumption, public consumption and gross fixed capital formation).

Table 1 shows various statistics directly obtained from the WIOD. In 2014 world GDP was 73, 806, 918 million US dollars, which was roughly 46 % of the world's total output (160,997,197 million US dollars). Also in Table 1, private consumption was the largest component of final demand, i.e., 41,998,603 million US dollars, which represented 56 % of the total amount (75,447,435 million US dollars).^17^ This was followed at great distance by investment (19,942,312 million US dollars), which represented 26 % of total demand. Public consumption (13,506,520 million), which accounted for the remaining 18 %, represented the lowest value.

**Table 1:** Aggregate data, 2014 (million US dollars).

*COUNTRY/REGION*	*PRODUCTION*		*FINAL DEMAND*			
	Value Added (GDP)	Output	Private Consumption	Public Consumption	Investment	TOTAL DEMAND
**China**	10,283,983	31,745,102	3,637,256	1,406,412	4,772,425	9,816,093
**United States**	17,348,070	30,971,023	11,908,807	2,556,267	3,432,623	17,897,697
**European Union**	13,924,330	29,077,129	7,423,623	3,487,357	2,850,372	13,761,352
**Rest of the World**	32,250,535	69,203,943	19,028,917	6,056,484	8,886,892	33,972,293
**TOTAL**	73,806,918	160,997,197	41,998,603	13,506,520	19,942,312	75,447,435

By country, the United States led value added (17,348,070 million US dollars), private consumption (11,908,807 million), and demand (17,897,697 million). China had the largest value for output (31,745,102 million) and investment (4,772,425 million), while the EU had the largest value for public consumption (3,487,357 million).

The figures in Table 1 illustrate the differences in the economic structures of these three areas. With regard to production, China had the highest output but the lowest value added. With regard to final demand, in the United States (where it represented roughly 28 % of the global value) private consumption was well above that of the other countries. Public consumption in the EU was clearly the highest, representing roughly 26 % of the world's total. Finally, China led the world in investment, where it was 65 % higher than in the EU and 39 % higher than in the United States.

## 4 Empirical application

### 4.1 Relative contribution to global GDP

Table 2 contains sectoral GDP (directly obtained from the WIOD) and the relative contributions of sectors and regions to the global amount (i.e., the elements calculated following the *product contribution vector* W in Equation (4)).

**Table 2:** Sectoral GDP and GDP contribution, 2014.

SECTOR		China		United States		European Union		Rest of the World		World	
		GDP^a^	$W_{1}$	GDP^a^	$W_{2}$	GDP^a^	$W_{3}$	GDP^a^	$W_{4}$	GDP^a^	W
1	Agriculture	979,397	0.01327	215,416	0.00292	245,896	0.00333	1,858,788	0.02518	3,299,497	0.04470
2	Coke and Petroleum	110,726	0.00150	170,173	0.00231	27,146	0.00037	243,354	0.00330	551,399	0.00747
3	Electricity and Gas	207,867	0.00282	271,532	0.00368	273,761	0.00371	799,284	0.01083	1,552,444	0.02103
4	Water	26,815	0.00036	52,289	0.00071	129,777	0.00176	211,822	0.00287	420,702	0.00570
5	Mining and Quarrying	564,306	0.00765	453,755	0.00615	76,296	0.00103	2,249,211	0.03047	3,343,568	0.04530
6	Food, Beverages and Tobacco	411,648	0.00558	245,013	0.00332	297,946	0.00404	742,820	0.01006	1,697,427	0.02300
7	Chemicals	301,256	0.00408	360,317	0.00488	276,476	0.00375	492,274	0.00667	1,430,322	0.01938
8	Metal Products	399,763	0.00542	205.931	0.00279	294,019	0.00398	634,632	0.00860	1,534,345	0.02079
9	Machinery and Equipment	689,291	0.00934	495,412	0.00671	603,924	0.00818	1,033,516	0.01400	2,822,144	0.03824
10	Motor Vehicles	308,412	0.00418	265,984	0.00360	295,441	0.00400	544,062	0.00737	1,413,899	0.01916
11	Textiles and Furniture	416,526	0.00564	162,340	0.00220	223,026	0.00302	578,857	0.00784	1,380,749	0.01871
12	Other Manufacturing	405,656	0.00550	215,039	0.00291	280,682	0.00380	580,373	0.00786	1,481,750	0.02008
13	Construction	702,036	0.00951	664,001	0.00900	725,795	0.00983	2,106,200	0.02854	4,198,032	0.05688
14	Trade and Commerce	1,003,854	0.01360	2,115,304	0.02866	1,543,674	0.02092	4,180,278	0.05664	8,843,110	0.11981
15	Land Transport	306,858	0.00416	239,714	0.00325	327,867	0.00444	1,124,165	0.01523	1,998,604	0.02708
16	Water Transport	59,481	0.00081	18,513	0.00025	39,302	0.00053	120,345	0.00163	237,641	0.00322
17	Air Transport	23,509	0.00032	84,044	0.00114	38,081	0.00052	108,938	0.00148	254,573	0.00345
18	Postal Activities	81,420	0.00110	163,413	0.00221	314,697	0.00426	376,164	0.00510	935,694	0.01268
19	Accommodation and Food services	200,016	0.00271	487,987	0.00661	393,441	0.00533	733,462	0.00994	1,814,907	0.02459
20	Publishing and Telecommunications	271,392	0.00368	1,074,200	0.01455	645,735	0.00875	1,243,938	0.01685	3,235,266	0.04383
21	Financial Services	623,873	0.00845	1,222,889	0.01657	693,765	0.00940	1,875,834	0.02542	4,416,360	0.05984
22	Education	339,284	0.00460	192,772	0.00261	712,402	0.00965	1,393,752	0.01888	2,638,211	0.03574
23	Human Health	187,320	0.00254	1,226,868	0.01662	1,045,741	0.01417	1,300,945	0.01763	3,760,873	0.05096
24	Public Administration	419,169	0.00568	2,274,607	0.03082	943,209	0.01278	2,068,163	0.02802	5,705,148	0.07730
25	Other Service Activities	1,244,110	0.01686	4,470,557	0.06057	3,476,230	0.04710	5,649,359	0.07654	14,840,256	0.20107
**TOTAL**		10,283,983	**0.13934**	17,348,070	**0.23505**	13,924,330	**0.18866**	32,250,535	**0.43696**	73,806,918	**1.00000**

a *Million US Dollars. Source*: Author's calculations based on the WIOD.

At the regional level (final row in Table 2), the United States (23.50 %) led contributions to global GDP. The US was followed by the European Union (18.86 %), while China had the lowest contribution (13.93 %) of the three regions. Joint contribution represented roughly 56 % of total production, which clearly confirms the agglomeration of world production in just a few geographical areas. Note that adding the contributions made by these three countries and ROW (43.69 %) provides total world GDP (100 % or 1.00 according to the model).

By sector, the right-hand column shows that the world's largest production was for Other Service Activities (Sector 25). This represents 20.1 % of total GDP, of which the US contributed 6.0 % and the EU 4.7 %. Also note Trade and Commerce (Sector 14), which accounted for 11.9 %. Jointly, these two activities represented roughly a third (32 %) of world production, which means that global GDP is largely dependent on these two service activities. Public Administration (Sector 24), Financial Services (Sector 21), Construction (Sector 13) and Human Health (Sector 23) also had notable contributions (7.7 %, 5.9 %, 5.6 %, and 5.0 %, respectively).

The first result to highlight from Table 2 is that not only is world GDP geographically concentrated but it is also asymmetrically distributed at the sectoral level. Another result is that China is at the forefront of world industrial production, with the highest contribution made by industries (Sectors 5 to 12). On the other hand, the United States leads the world's services production (Sectors 14 to 25), except in Sector 22 (Education), which is led by the European Union.

### 4.2 Changes in contribution to global GDP

This section illustrates the changes in relative GDP by focusing on the effects that modifying the model's (exogenous) final demand has on contributions to global GDP by sectors and countries.

Table 3 summarizes the results of an exogenous change in final demand. This change can come, for instance, from a rise in private income that increases private consumption, an increase in public expenditure, or an increase in investment by sectors. Since this simulation evaluates the impact on relative contribution to GDP that was originated in final demand, it can be interpreted as an evaluation of how cyclical fluctuations of demand affect the importance of sectors and countries within world production. Understanding the relationship between demand shocks and relative GDP will improve our knowledge of the consequences of the economic cycle at both the world level and the regional (i.e., country) level.

**Table 3:** GDP reallocation under unitary and exogenous changes in final demand, 2014: Regions^a^.

*COUNTRY/REGION*	China	United States	European Union	Rest of the World	World TOTAL
**China**	18.62	−3.21	−3.03	−2.79	9.58
**United States**	−5.60	16.39	−5.05	−5.04	0.70
**European Union**	−4.36	−4.27	15.92	−3.59	3.70
**Rest of the World**	−8.65	−8.91	−7.84	11.42	−13.98
**TOTAL**	0	0	0	0	0

a *Million US Dollars. Source*: Author's calculations based on the WIOD.

The figures in Table 3 correspond to the regionally aggregated elements of matrix e’GZ. This calculation transforms the reallocation process (matrix Z) in order to keep the amount of GDP constant at the initial level. In other words, since the changes in the relative contribution (Z) are pre-multiplied by total GDP (e’G), the resulting matrix can be interpreted as the reallocated GDP for all the bilateral elements captured by the model. Since the columns in this matrix also add up to zero, the reflected process can be seen as a compensating mechanism of winners and losers in net terms.^18^

The values in Table 3 should be interpreted as follows. When China receives an exogenous and unitary demand inflow, its relative GDP increases by 18.62 million US dollars, whereas the impact on the other regions is negative. In particular, the impact on the United States is a decrease of 5.60 million dollars, and the impact on the European Union and the Rest of the World is, respectively, a decrease of 4.36 and 8.65 million dollars.

The columns in Table 3 contain the redistributed GDP when an exogenous shock in demand is received by the country in the column. Interestingly, all values in this table are negative except the symmetrical elements. This indicates that the bilateral relations between regions are detrimental in terms of the impacts they receive on the relative product, and only inflows received domestically and materialized in domestic input-output transactions can improve the contribution to global GDP.

The rows in Table 3 show the changes in the relative GDP of the region in the row when they all simultaneously receive a unitary inflow in final demand. Interestingly, we can see from the right-hand column that the highest (positive) change is for China (9.58 million dollars), which means that increases (decreases) in the final demand of all countries would increase (decrease) Chinese contribution to global GDP. This is achieved at the cost of decreasing the contribution from the Rest of the World, which shows a large negative value (−13.98 million dollars). The EU, and to a lesser extent the US, have positive values in the right-hand column (3.70 and 0.70 million, respectively). In summary, Table 3 indicates that an economic expansion (crisis) that increases (reduces) world demand, would increase (reduce) the importance of China at the global level and reduce (increase) the contribution from the Rest of the World. Importantly, while the European Union and the United States evolve in the same direction as China, the magnitude of the redistribution in these two areas is much lower than the impact received by China.

Table 4 shows the sectoral decomposition of the right-hand column in Table 3. The figures in this table quantify how many dollars of GDP are reassigned among sectors of production when total GDP is held constant at the initial level and final demand in all regions increases by one monetary unit. For instance, when there is a generalized and unitary inflow in world demand, Agriculture (Sector 1) increases its relative GDP by 0.75 million dollars in China, by 0.56 million dollars in the United States, and by 0.42 million dollars in the European Union. On the other hand, the agricultural sector in the Rest of the World decreases its relative GDP by 0.87 million dollars. This results in a global impact received by agriculture that amounts to 0.85 million dollars of relative GDP after a generalized increase in world demand. The rest of the figures in Table 4 should be read in a similar way.

**Table 4:** GDP reallocation under unitary and exogenous changes in final demand, 2014: Sectors^a^.

SECTOR		China	United States	European Union	Rest of the World	World TOTAL
1	Agriculture	0.75	0.56	0.42	−0.87	0.85
2	Coke and Petroleum	0.30	0.27	0.07	0.12	0.75
3	Electricity and Gas	0.48	0.54	0.43	0.04	1.49
4	Water	0.40	0.58	0.48	0.39	1.84
5	Mining and Quarrying	1.40	1.01	0.67	1.66	4.74
6	Food, Beverages and Tobacco	0.25	0.11	0.06	−0.47	−0.05
7	Chemicals	0.39	0.46	0.37	0.12	1.34
8	Metal Products	0.21	0.47	0.40	0.03	1.10
9	Machinery and Equipment	0.04	0.25	0.12	−0.29	0.12
10	Motor Vehicles	0.14	0.10	0.07	−0.17	0.13
11	Textiles and Furniture	0.17	0.32	0.25	−0.15	0.59
12	Other Manufacturing	0.25	0.37	0.37	−0.01	0.98
13	Construction	−0.61	−0.18	−0.23	−2.12	−3.14
14	Trade and Commerce	0.69	−0.99	−0.23	−2.85	−3.38
15	Land Transport	0.63	0.46	0.51	−0.35	1.26
16	Water Transport	0.47	0.29	0.25	0.39	1.39
17	Air Transport	0.30	0.41	0.25	0.26	1.21
18	Postal Activities	0.52	0.73	0.76	0.42	2.43
19	Accommodation and Food services	0.35	0.04	0.12	−0.31	0.19
20	Publishing and Telecommunications	0.51	−0.24	0.21	−0.50	0.00
21	Financial Services	1.12	−0.18	0.35	−0.78	0.51
22	Education	0.19	0.38	−0.03	−1.04	−0.49
23	Human Health	0.11	−1.01	−0.64	−1.10	−2.63
24	Public Administration	0.04	−1.98	−0.35	−1.94	−4.23
25	Agriculture	0.48	−2.04	−0.98	−4.45	−6.99
**TOTAL**		**9.58**	**0.70**	**3.70**	**−13.98**	**0**

a *Million US Dollars. Source*: Author's calculations based on the WIOD.

For China, all sectoral values are positive except Construction (Sector 13), which reduces its relative GDP by 0.61 million dollars after a unitary increase in demand. The signs of GDP reallocation for sectors in the United States and the European Union follow a similar pattern, though the European values are generally higher than those of the United States. In particular, these two regions show reductions in the relative GDP for Construction (Sector 13), Trade and Commerce (Sector 14), as well as various service activities. Table 4 also shows that Agriculture (Sector 1), various industrial activities, Construction (Sector 13), and most services in the Rest of the World suffer reductions in their relative contribution to world GDP.

Finally, the right-hand column in Table 4 shows the changes in the relative GDP of sectors at the world level. This is an interesting outcome for international economics, since these values illustrate which sectors gain importance and which ones lose importance within global production. Among the positive impacts, Electricity and Gas (Sector 3), Water (Sector 4), Chemicals (Sector 7), Metal Products (Sector 8), and Transport Services (Sectors 15, 16, 17, and 18) show a considerable increase. On the other hand, there are clear reductions in relative GDP for Construction (Sector 13), Trade and Commerce (Sector 14), Public Administration (Sector 24) and Other Services (Sector 25).

### 4.3 Discussion of the results

Previous literature reported a persistent and growing gap between rich and poor countries across the world during the second half of the twentieth century. In addition, in developing countries the gap in personal income distribution, specifically between the rich and the poor, also increased. In short, previous research established the generally accepted wisdom that both internal and international income gaps were widening. This line of knowledge used empirical analyses that employed econometric and statistical approaches to determine a persistent difference between the rich and the poor [3]. This assertion represented a rebuttal to the economic convergence hypothesis [4], which suggests that the phenomenon of rich and poor disparity is transitory and can be reduced. This literature also described a downturn trajectory in the differences in global income distribution since the turn of the century that was largely explained by the rapid growth in certain Asian countries (i.e., India and China), though the within-country gap continued to increase [5].

The results reported in the present study contribute to this evidence by clearly suggesting that a universal economic expansion is linked to a boost in the asymmetries of production across the world and that a global economic crisis would reduce the differences in the production worldwide. In other words, these findings suggest that economic growth is linked to a concentration of world production in countries with the highest initial GDP (particularly China and, to a lesser extent, countries of the European Union). These outcomes reinforce the argument that, in marked contrast to the convergence theory, the distance between the GDP in rich and poor countries is increasing.

To gain knowledge of the processes that determine global production and their implications for individual countries and regions, it is crucial to disentangle the underlying mechanisms governing the current geography of world production. The role individually played by countries within the global economic system has significant consequences for national production, domestic welfare and the capacity to exert economic influence in world markets. The ability to uncover the mechanisms that affect relative individual positions within the complexity of world production is therefore crucial to defining redistribution measures worldwide. It is also useful to anticipate and counterbalance the automatic reallocation mechanisms that can harm internal economic activity and domestic income. Moreover, all these findings have important policy implications globally since they support the idea of a diverging economic trend across the world and reaffirm the postulate that policy responses to economic divergence should play a role in slowing the gap between rich and poor economies. The outcomes in this paper should therefore guide key aspects of international economics such as trade and monetary agreements or environmental protocols.

Since this paper proposes a novel input-output modelization of world production for analyzing distribution mechanisms, it can be considered complementary to previous knowledge, which was mainly based on statistical and econometric methods. However, it is important to bear in mind that since the input-output production structure does not allow substitution possibilities, the results appear in an upper range of possible impacts. It is also worth noting that the model used identifies winners and losers in net terms since the relative income determination necessarily implies a zero-sum game in which total relative increases in the individual contribution to world GDP are equal to total relative decreases. Despite these weaknesses, this framework has undeniable advantages, namely its faithful connection with the economic complexity involved and its ability to provide detailed information. All these aspects make the input-output model a powerful tool for analyzing global production issues.

## 5 Conclusions

Economic globalization has concentrated production in a limited number of countries and regions around the world. Indeed, adapting production processes to a globally organized setting has led to substantial changes in economic geography so that nowadays just a few regions are driving the world's entire GDP.

This paper provides a novel framework for calculating the contributions of countries to global production. By defining a simple and treatable general equilibrium structure for world GDP, we can evaluate the extent to which the relative contributions of countries/regions to global production are affected by changes in the model's (exogenous) final demand. The method proposed contributes to the debate on the gap between rich and poor countries as it shows whether this gap decreases (i.e., the economies converge) or increases (i.e., the economies diverge) when an increase in demand is produced. In turn, this sheds light on the ability of the automatic mechanisms operating in world production to modify inequalities across countries.

Several interesting aspects from this paper deserve to be highlighted. First, a limited number of service sectors monopolize the largest contribution to global GDP. This sectoral concentration of product into a few activities warrants special attention from the field of international economics given its potential implications for income generation and income distribution around the world. Second, the outcomes presented in this paper provide new insights into the impacts that shocks in demand cause in the relative contributions of countries to global production. In particular, China's relative GDP increases after an increase in world demand at the expense of the ROW's relative GDP. This suggests that economic growth *per se* does not reduce the gap between rich and poor countries since the most important producers (i.e., those with the highest contribution to world GDP) are receiving the greatest impacts on their relative production. Moreover, it should be pointed out that all the bilateral country-to-country effects are negative and that only demand shocks occurring and materialized domestically have a positive impact on relative GDP. Third, this model can be easily applied at the empirical level by using available global inter-country input-output statistics. In addition, since this method provides a novel tool for evaluating the contributions of individual countries to global GDP, it may be useful for improving decision-making in areas such as the geopolitics of production, economic cycle theory and world economic planning.

Finally, although the framework proposed provides new information about GDP determination worldwide, the potentialities of the analysis go beyond the application shown in this article. Many extensions to this approach can be used to analyze other interesting issues by defining variables other than world GDP, such as world employment and global emissions. Since the information required can be obtained from available global databases, such extensions can be applied easily.

## Data Availability statement

Data used in this article are available at www.wiod.org.

## Ethics statement

Informed consent was not required for this study because no personal or individual data were used.

## CRediT authorship contribution statement

**Maria Llop:** Writing – review & editing, Writing – original draft, Validation, Methodology, Formal analysis, Data curation, Conceptualization.

## Declaration of competing interest

The author declares no relevant or material financial interests that relate to the research described in this paper. The author also declares no conflict of interest.

## Footnotes

1 This group comprises 21 countries, including the United Kingdom.

2 [2] first discussed the gap between rich and poor economies and examined several factors that may explain it. See Ref. [3] for a recent description of this gap.

3 [4] exhaustively examined the growth and convergence theories developed so far.

4 [5] provides a recent analysis of global and regional income distribution during the period 2008–2013. Additionally [6,7], show the relationship between the countries' income per capita and well-being by paying special attention to the possible limitations of using income as a measure of welfare. Additionally [8], compared GDP and its components for 176 countries by reviewing several important measures, such as health and international transfers of capital and property rights.

5 In a recent contribution [9], revisits the relationship between trade and GDP and presents a large cross-country comparison. Also see [10] for an examination of the relationship between GDP and the international trade of nations across continents and worldwide.

6 For example [11], evaluates the connection between the economic growth of emerging economies and their representation in various international institutions. Additionally [12], discusses the usefulness and limitations of the GDP as a metric of the economic success of countries.

7 Among these contributions, see for instance [13,14], and [15].

8 See, for example [[16], [17], [18], [19]], and [20].

9 See, for example, [21].

10 [22] provide a conceptual definition of the multiregional model and describe the progress made so far in this branch of input-output literature.

11 For examples of applications, see those by [26] for China's provinces [27], for the emissions embodied in China-United States trade [28], for European Union countries, and [29] for a Japanese metropolitan region.

12 See [31] for relative income determination in the SAM model.

13 In what follows, the multiregional model is assumed to include all regions of the world. This implies that g is global (world) product.

14 See [37] for details on the construction and structure of this database.

15 The European Union comprises the 27 countries that are currently part of the Union.

16 Note that, as China and the United States are countries included in the analysis, the data for these countries are the same as in the original database, with the only difference being in the sectoral detail.

17 According to the WIOD, the amount of world value added (73, 806, 918 million dollars) is not exactly equal to world final demand (75,447,435 million dollars) because of international transport margins, taxation on products and other accounting adjustments.

18 It can be checked that the columns of e’GZ add up to zero, as do those of Z (e’(e’GZ)=0).
